# A predictive model for identifying patients at risk of delayed transfer of care: a retrospective, cross-sectional study of routinely collected data

**DOI:** 10.1093/intqhc/mzab130

**Published:** 2021-09-06

**Authors:** Andrew Davy, Thomas Hill, Sarahjane Jones, Alisen Dube, Simon c Lea, Keiar l Watts, M d Asaduzzaman

**Affiliations:** University Hospitals of North Midlands NHS Trust, Royal Stoke University Hospital, Newcastle Road, Stoke-on-Trent ST4 6QG, UK; University Hospitals of North Midlands NHS Trust, Royal Stoke University Hospital, Newcastle Road, Stoke-on-Trent ST4 6QG, UK; Department of Engineering, School of Digital, Technologies and Arts, Staffordshire University, Room B009A, Cadman Building, Stoke on Trent ST4 2DE, UK; Department of Engineering, School of Digital, Technologies and Arts, Staffordshire University, Room B009A, Cadman Building, Stoke on Trent ST4 2DE, UK; University Hospitals of North Midlands NHS Trust, Royal Stoke University Hospital, Newcastle Road, Stoke-on-Trent ST4 6QG, UK; University Hospitals of North Midlands NHS Trust, Royal Stoke University Hospital, Newcastle Road, Stoke-on-Trent ST4 6QG, UK; Department of Engineering, School of Digital, Technologies and Arts, Staffordshire University, Room B009A, Cadman Building, Stoke on Trent ST4 2DE, UK

**Keywords:** delayed transfer of care (DTOC), predictive modelling, mixed-effect logistic regression, ROC curve, sensitivity and specificity

## Abstract

**Background:**

Delays to the transfer of care from hospital to other settings represent a significant human and financial cost. This delay occurs when a patient is clinically ready to leave the inpatient setting but is unable to because other necessary care, support or accommodation is unavailable. The aim of this study was to interrogate administrative and clinical data routinely collected when a patient is admitted to hospital following attendance at the emergency department (ED), to identify factors related to delayed transfer of care (DTOC) when the patient is discharged. We then used these factors to develop a predictive model for identifying patients at risk for delayed discharge of care.

**Objective:**

To identify risk factors related to the delayed transfer of care and develop a prediction model using routinely collected data.

**Methods:**

This is a single centre, retrospective, cross-sectional study of patients admitted to an English National Health Service university hospital following attendance at the ED between January 2018 and December 2020. Clinical information (e.g. national early warning score (NEWS)), as well as administrative data that had significant associations with admissions that resulted in delayed transfers of care, were used to develop a predictive model using a mixed-effects logistic model. Detailed model diagnostics and statistical significance, including receiver operating characteristic analysis, were performed.

**Results:**

Three-year (2018–20) data were used; a total of 92 444 admissions (70%) were used for model development and 39 877 (30%) admissions for model validation. Age, gender, ethnicity, NEWS, Glasgow admission prediction score, Index of Multiple Deprivation decile, arrival by ambulance and admission within the last year were found to have a statistically significant association with delayed transfers of care. The proposed eight-variable predictive model showed good discrimination with 79% sensitivity (95% confidence intervals (CIs): 79%, 81%), 69% specificity (95% CI: 68%, 69%) and 70% (95% CIs: 69%, 70%) overall accuracy of identifying patients who experienced a DTOC.

**Conclusion:**

Several demographic, socio-economic and clinical factors were found to be significantly associated with whether a patient experiences a DTOC or not following an admission via the ED. An eight-variable model has been proposed, which is capable of identifying patients who experience delayed transfers of care with 70% accuracy. The eight-variable predictive tool calculates the probability of a patient experiencing a delayed transfer accurately at the time of admission.

## Introduction

When patients are admitted to hospitals, it is important that they are discharged in a timely manner once they no longer require the expertise that is provided by the clinical team. A delayed transfer of care (DTOC) occurs when an adult inpatient is medically ready to go home but is still occupying a hospital bed [1]. Delays to discharge can have serious implications such as mortality, infections, depression and reductions in patients’ mobility and their ability to undertake daily activities [2]. In February 2020, there were a total of 155 700 delayed days, equivalent to an average of 5370 people delayed per day in the National Health Service (NHS) in England [3]. This phenomenon is not specific to England, it is an international challenge [4–6].

The cost due to discharge delay among patients aged 65 and over has been estimated at £820 million for the period 2013–14. The most recent data (for 2019) show that 1.7 million bed-days were lost, with an associated cost of over £1 billion [7, 8]. These delayed discharges are referred to as ‘bed-blocking’ in the literature and ‘DTOCs’ by the NHS, have been persistently occurring within the NHS secondary care setting [9]. Bed-blocking or DTOCs primarily affect those patients directly waiting to be discharged from hospitals, but there is also a significant secondary effect on those patients who are waiting for admission from emergency portals in the hospital. These patients cannot move from the emergency portals to the wards until the current patients on the wards are discharged from these ward beds to their homes. This bottleneck effect on flow causes significant overcrowding within emergency departments (EDs) and other emergency portals, which results in increased mortality, poor patient outcome and significantly higher consumption of hospital resources [10, 11]. DTOC also causes uncertainty and distress to patients and their families. Moreover, it increases the already significant pressure on the care systems and reduces the system capacity [9].

The problem of delayed discharges continues, despite major changes in the provision and organization of health and long-term care services, including various measures to improve integration between the sectors. Delayed discharge has been one of the major policy concerns for the NHS [12]. The House of Commons Health Committee report shows that delayed discharge is one of the major reasons preventing hospital accident and EDs from achieving their target of 95% of patients being admitted, transferred or discharged within 4 hours [13].

One of the main targets in the NHS Long-Term Plan is ‘cutting delays in patients being able to go home’ [12]. The report states that the goal is to keep the average number of patients DTOC per day to 4000 or below with an aim to reduce it further in upcoming years [12]. The Long-Term Plan also envisaged that the performance of the NHS and social care would improve by reducing unnecessary delays for patients when they are ready to be discharged from hospitals.

The Better Care Fund initiative was launched to support relevant organizations to work closely together to reduce delays in discharge and provide integrated health and social care services across England to achieve the vision outlined in the Long-Term Plan [12, 14]. With around 18% of the population in the UK being elderly (65 years and over), the NHS has been struggling to reduce the delays of discharge despite these initiatives. Exploiting advanced statistical modelling, data dashboards and communication technologies, it is foreseeable that complex problems like delays of discharge can be ameliorated. Due to the inherent complexities involved in delayed transfers of care, there have been very few attempts to model and identify related factors [15, 16]. On review, these two studies investigated the clinical and social factors of DTOC. These factors included availability of hospital beds, care pressures, availability of home hospitalization unit, psychosocial factors including available family members, living alone and living in a nursing home. However, this information has not been translated into a predictive model.

In this study, we investigate routinely collected data to identify risk factors related to DTOC for patients admitted to hospital following attendance at the ED. The data used are pragmatic in terms of ease of access and availability, and provide a reasonable strategy in developing a working model. Using routinely collected administrative data with some clinical information, we investigated the potential to develop a predictive model for identifying those patients most at risk of DTOC on discharge. Such a model could enable early identification of patients with potential inhibitory factors to a timely discharge. The prediction would help to make sure that patients have timely therapy review, or enable community teams to be sighted early on expected care plan needs. We believe that this would help better discharge planning locally and across the wider NHS. This would also facilitate the NHS to meet its strategic priorities, including reduction of workforce burden, improvement of safety across health and care systems, and increase health and care productivity by utilizing digital technology. Being able to predict, when the patient is first admitted, those most at risk of a DTOC upon discharge, could facilitate earlier discharge planning and improve the discharge pathway.

## Methods

### Study design

This is a single centre, retrospective, cross-sectional study of routinely collected hospital administration and clinical data.

### Setting and participants

The setting for this study was the Royal Stoke ED of University Hospitals of North Midlands NHS Trust (UHNM), an NHS organization in the Midlands, England. The hospital has approximately 1100 beds and experiences approximately 95 000 attendances annually based on the Medway Database of the hospital. Of these attendances, approximately 48% result in admission to the hospital.

Routinely collected available data relating to the Type 1 ED attendance and subsequent admission were retrieved for the period 1 January 2018 to the 31 December 2020, including a 9-month period covering the Covid-19 pandemic in England. Anonymized data were extracted from the hospital patient administration system and exported into a Microsoft Excel format. Data pertaining to all patients aged over 18 years who had been admitted to the hospital following an ED admission were eligible for inclusion during the study period and retrieved as part of the data extraction process.

### Variables

All data were retrieved from a single database that warehouses routinely collected clinical and administrative data. Following an attendance at the ED, the final clinical decision was categorized as either admission or non-admission. In this study, admissions following an ED attendance were investigated to ascertain the risk of a subsequent DTOC. DTOC data are collected routinely as part of standard patient care in England and form the outcome variable of interest in this study.

Information on several potential risk factors for DTOC are recorded throughout the patient journey from triage to treatment and discharge. In this study, age, gender, ethnicity, the national early warning score (NEWS), triage category (three categories), referred by the GP, arrived by ambulance, admitted in the last 12 months, the Glasgow admission prediction score (GAPS) and the index of multiple deprivation (IMD) were investigated for possible association with, and identification of patients at risk of DTOC. These variables are recorded at the time of admission and were found to be potentially correlated with the DTOC (see Table 1).

**Table 1:** Characteristics of training and validation datasets

	Overall dataset	Training data			Test data	
	**Total** ***n* = 134 231**	**Non-DTOC (*n* = 85 245)**	**DTOC** **(*n* = 8543)**		**Non-DTOC** **(*n* = 36 800)**	**DTOC** **(*n* = 3643)**
Variables	**Mean, SD range**	**Mean, SD range**	**Mean, SD range**	**Effect size, *p*-value and 95% CI^a^**	**Mean, SD range**	**Mean, SD range**
Age (years)	63, 21 (18, 120)	61, 21 (18, 120)	81, 10 (18, 105)	Mean diff. = −20.14 CI (−20.40, −19.88) <0.0001	61, 21 (18, 119)	81, 11 (18, 105)
Gender^b^						
Male (%) Female (%)	64 407 (48.0) 69 768 (52.0)	41 398 (48.6) 43 816 (51.4)	3500 (41.0) 5039 (59.0)	OR (male ref.) = 1.36 <0.0001	18 065 (49.1) 18 714 (50.9)	1444 (39.6) 2199 (60.4)
Ethnicity^c^						
Caucasian Others	122 750 (96.2) 4945 (3.8)	77 596 (95.8) 3377 (4.2)	8181 (99.0) 82 (1)	OR (ref. Others) = 4.34 <0.0001	33 458 (95.8) 1455 (4.2)	3515 (99.1) 31 (0.9)
GAP score	24, 8 (6, 57)	24, 8 (6, 56)	27, 7 (10, 57)	Mean diff. = −3.64 CI (−3.81, −3.48) <0.0001	24, 8 (6, 57)	27, 7 (11, 52)
IMD^d^	5, 3 (1, 10)	4, 3 (1, 10)	5, 3 (1, 10)	Mean diff. = −0.19 CI (−0.26, −0.13) <0.0001	4, 3 (1, 10)	5, 3 (1, 10)
NEWS	1.6, 2.4 (0, 19)	1.6, 2.4 (0, 18)	2.1, 2.7 (0, 16)	Mean diff. = −0.53 CI (−0.59, −0.47) <0.0001	1.6, 2.4 (0, 19)	2.1, 2.7 (0, 17)
Triage category						
Immediate Urgent Non-urgent	61 882 (46.1) 52 491(39.1) 19 758 (14.8)	39 526 (46.4) 33 383(39.2) 12 336 (14.4)	3816 (44.7) 3412 (39.9) 1315(15.40	OR (ref. Non-urgent) 1.06 1.10 <0.001	17 046 (46.3) 14 209 (38.6) 5545 (15.1)	1594 (43.8) 1487 (40.8) 562 (15.4)
Arrived by ambulance						
Yes No	97 408 (72.6) 36 823 (27.4)	59 857 (70.2) 25 388 (29.8)	8283 (97.0) 260 (3.0)	OR (ref. No) = 13.51 <0.0001	25 771 (70.0) 11 029 (3.0)	3497 (96.0) 146 (4.0)
Admitted in the last 12 months						
Yes No	72 048 (53.7) 62 183 (46.3)	44 745 (52.5) 40 500 (47.5)	5799 (67.9) 2744 (32.1)	OR (ref. No) = 1.91 <0.0001	19 090 (51.9) 17 710 (48.1)	2414 (66.3) 1229 (33.7)
GP referral						
Yes No	12 711 (9.5) 121 520 (90.5)	8594 (10.1) 76 651 (89.9)	274 (3.2) 8269 (96.8)	OR (ref. No) = 0.30 <0.0001	3743 (10.2) 33 057 (89.8)	100 (2.7) 3543 (97.3)

a–based on training dataset only using either Mann–Whitney U Test or Chi-squared tests as appropriate;

b 0.04% missing data,

^c^ 4.87% missing data,

^d^ 1.40% missing data.

### Statistical analysis

Statistical analysis was carried out in R [17] and STATA 14 [18]. Data from all patients aged over 18 years who had been admitted to the hospital through the ED between the 1 January 2018 and the 31 December 2020 were randomly allocated to either the training dataset (70%) or the validation dataset (30%). However, there have been significant changes in hospital admissions and discharges due to Covid. As the standard practice, data were randomly allocated into training and test datasets that would also help to reduce the bias due to Covid.

The following variables were found to have missing data: gender, 0.04%; ethnicity, 4.87%; and IMD decile, 1.40%. In total, 6% of patients had missing data. As the proportion of missing data is below 10% and is considered to be within acceptable parameters [19, 20], they were discarded from the model development and validation dataset.

A mixed-effects logistic regression model was used to identify predictors of DTOCs, which takes into account patients with multiple admissions within the study period [21]. Due to the dichotomous nature of the dependent variable, the logistic regression was chosen, and the mixed-effects model allowed to capture the multiple admissions of the same patient introducing a patient-specific random effect. Stepwise backward elimination was applied to identify the final variables to be included in the model [22]. Only variables that showed a significant effect (*p* < 0.05) were included in the final model. The final model selection was based on the Akaike information criterion values; odds ratios and 95% confidence interval (CI) were also calculated. The performance of the predictive model was assessed using the area under the receiver operating curve (ROC AUC) using the validation dataset only [22]. The AUC can range from 0.5 to 1. An AUC of 0.5–0.7 is interpreted as a model with low discriminatory power, 0.7–0.9 as moderate and >0.9 as a model with a high discriminatory power [23].

## Results

A total of 134 231 admissions following an ED attendance were retrieved over the three-year study period. Of these, 12% of patients (16 224/134 231) had more than one admission. Patients with multiple admissions had an average of 2.93 (SD = 1.92, range = 2, 54) admissions per patient. Less than 2% had missing data for either gender or ethnicity and less than 5% for IMD score variable as a result overall 6% data were found to be missing. Therefore, a total of 132 321 admissions had complete data and were included in the analysis; 93 788 (70%) admissions were included in the training dataset and 40 443 (30%) admissions in the validation dataset. Nine percent of the admissions in each of the datasets experienced a DTOC. The overall study population had a mean age of 63 years (SD = 21; range = 18, 120) and 52% (69 768/132 321) were female. Over 90% (121 189/132 321) were Caucasian and at least 54% had an IMD score of four or less.

### Predictive modelling using mixed-effects logistic regression

The 10 variables considered for the predictive model are presented in Table 1. These variables were chosen because they were clinically felt to be significant and were routinely collected information or in the case of IMD data, was open-source and therefore easily obtainable. Of these ten variables we reviewed, eight were found to be statistically significant in predicting DTOC. Age, gender, ethnicity, GAPS, IMD score, NEWS, mode of arrival and previous admission status (within the last 12 months) were included in the final model and their respective regression parameters are reported in Table 2. Patients that arrived by ambulance were at least five times more likely to result in a DTOC compared to those that arrived by other modes of transport. An increase in age and NEWS scores increased the odds of experiencing a DTOC. Furthermore, being Caucasian or female increased the odds of experiencing a DTOC, whilst being less deprived and having a low GAP score reduced the odds.

**Table 2:** Multiple logistic regression analysis results for the final prediction model

Variables	Estimated coefficient	*p*-value	OR (95% CI)
Age	0.074	<0.001	1.08 (1.07, 1.08)
Gender (ref. cat: Male)	0.198	<0.001	1.22 (1.15, 1.30)
Ethnicity (ref. cat: Others)	0.475	0.001	1.61 (1.22, 2.11)
GAP score	−0.009	0.001	0.991 (0.986, 0.996)
IMD	−0.027	<0.001	0.97 (0.96, 0.98)
NEWS	0.027	<0.001	1.03 (1.01, 1.04)
Arrival by ambulance (ref. cat: No)	1.750	<0.001	5.76 (5.01, 6.61)
Admitted in last 12 months (ref. cat: No)	0.365	<0.001	1.44 (1.35, 1.54)

**Figure 1:**
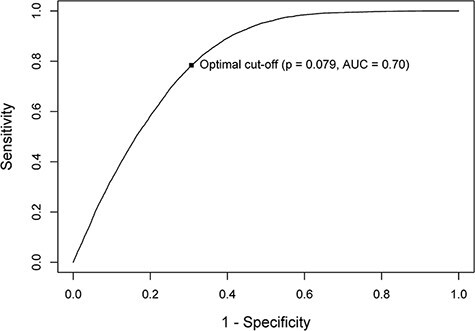
Receiver operating characteristic curve in predicting DTOC patients.

The predictive model achieved sensitivity and specificity of 0.77 (95% CI 0.75, 0.78) and 0.70 (95% CI 0.69, 0.70) respectively, using an optimal cut-off probability of 0.08. The predictive model yielded an AUC 0.70 (95% CI 0.69, 0.71) using the validation dataset, which indicates that the model had moderate discriminatory power (see Figure 1).

## Discussion

### Statement of principal findings

Age, gender, ethnicity, NEWS, GAPS, IMD decile, arrival by ambulance and admission within the last year were found to have a statistically significant association with delayed transfers of care. Patients who arrived by ambulance were 13 times more likely to experience DTOC. Arrival by ambulance suggests a more clinically ill patient, someone with less physical or mental ability to attend through other modes of transport, or someone arriving from a nursing home. The proposed eight-variable predictive model showed good discrimination with 79% sensitivity (95% CIs: 79%, 81%), 69% specificity (95% CI: 68%, 69%) and 70% (95% CIs: 69%, 70%) overall accuracy of identifying patients who experienced a DTOC. The model can be used to inform discharge planning at the point of admission.

### Strength and limitations

The key strength of our study is that we applied a mixed-effect logistic regression method on routinely collected data to identify patients at risk of DTOC. The proposed predictive model showed good discrimination with 79% sensitivity, 69% specificity and 70% overall accuracy of identifying patients who experienced a DTOC. However, this study represents patients attending and being admitted to a single ED and therefore, may not be transferrable to other organizations. However, it provides a point of comparison for other studies to draw on if investigating the risk factors for DTOCs and trying to model their predictive values. For this study, the whole adult admission population, representing 132 321 admissions, was investigated—the largest study of its kind.

The Covid-19 pandemic would have inevitably affected data pertaining to the period March 2020 to December 2020, but this is mitigated by having three years of data. For large periods during this time, commencing on the 23 March 2020, citizens of England were legally required to ‘stay home’ [24]. Whilst this did not exclude access to healthcare, patients may have been more reluctant to attend hospital. Therefore, it is possible that those patients seen during this time were more clinically unwell and this may have skewed the admission profile of patients.

In order to combat some of these limitations, a multi-centre study would serve to increase generalizability. To improve the moderate discriminatory power, more clinical data could be retrieved from different data sources. Incorporating this data could yield greater accuracy, for example, although seldomly recorded at present, when it was recorded ‘living alone’ was noted to double a patient’s risk of having a DTOC.

However, a key underlining purpose to this initial work for the clinicians at the hospital was to ascertain whether DTOCs could be predicted at the transition point from the ED to the ward in order to facilitate more appropriate discharge with currently available information to facilitate a real-time assessment of risks for patients as they are admitted.

Aligning and combining datasets for research purposes would yield better results. With the expected improvement in integrated care records, it is entirely foreseeable that patient data pertaining to frailty scoring, comorbidity and medication burden as well as domiciliary status (lives alone) could significantly improve the predictive power of this model.

### Interpretation within the context of the wider literature

This three-year retrospective observational study of routinely collected data investigated predictors for DTOC at the point of admission for all adult ED attendances during the investigation period. Other studies have tended to focus on elderly populations specifically and on a given ward presenting a new opportunity to understand risk factors [16]. A smaller, shorter study [15] investigating elderly admissions has previously shown that clinical frailty is a predictor of DTOC. Arrival by ambulance could represent a proxy for frailty in this study. Unfortunately, frailty scores are not yet routinely electronically stored for all admissions, but this is an area to explore in terms of refining the model and increasing accuracy. Moore et al’s [16] study showed that social factors contributed to the delayed discharge of elderly patients, citing delays to the arrangements of social care support to manage the functional decline typically experienced in elderly patients, as conducive to the delay.

### Implications for policy, practice and research

With an ageing population, DTOCs represent a growing risk for inappropriate care delivery, where patients are not receiving the optimal type of care for their needs. These patients are amongst some of the most vulnerable, disabled and frail and whilst clinical care in a hospital might not be necessary, they are likely to need significant other support to enable a high quality of independent or supported living. Understanding which patients are most statistically likely to experience a DTOC, could help target patients for proactive supportive discharge planning early on in their care journey.

It is envisioned that this could be achieved by alerting internal teams like therapists (Occupational therapists and physiotherapists) early on regarding high-risk patients thus enabling better workforce and case-load planning. It would also reduce time-lags in brokering external partners in the healthcare systems integral to the discharge planning process, such as social care, enabling more timely provision of community care plans and placements in residential and nursing homes.

## Conclusion

Delayed transfers of care have significant human costs and financial implications. Understanding the risk factors and then using these to predict DTOC patients if communicated with both internal and external care teams provides an opportunity to facilitate better resource planning and timely discharge. The aim of this early identification of at-risk patients would be to improve both the patient journey of those patients directly affected by delayed discharge but also those indirectly unable to move into the internal bed base because of this. By prioritizing those highlighted as being at risk of DTOC for an early review opportunity is created to facilitate their timely discharge with the inherent benefit to patients described above and a significant reduction of health care costs to the system. In future studies, we would like to include more potential factors such as availability of beds, home hospitalization units and their types, care pressure, the patients frailty score, multimorbidity and high levels of prescribing. The use of these with machine learning techniques could improve the accuracy of prediction and increase the practice value of such a model to clinicans. Where these data may not be routinely collected, their corresponding proxy measures may be used.

## Data Availability

No new data were generated or analysed in support of this article.
